# Lycopene protects against ionizing radiation‐induced testicular damage by inhibition of apoptosis and mitochondrial dysfunction

**DOI:** 10.1002/fsn3.3794

**Published:** 2023-10-30

**Authors:** Mingyue Qu, Qican He, Baoshi Guo

**Affiliations:** ^1^ Department of Medical Research The PLA Rocket Force Characteristic Medical Center Beijing China

**Keywords:** apoptosis, ionizing radiation, lycopene, oxidative stress, testis

## Abstract

Ionizing radiation (IR) is one of the key contributors that cause male infertility by disturbing spermatogenesis. Lycopene, a carotenoid with strong antioxidant properties, was shown to protect against oxidative damage induced by IR in several experimental models. The present study was designed to explore the possible protective effects of lycopene against IR‐induced testicular damage in C57BL/6 mice. Mice were administered lycopene (20 mg/kg) by oral gavage for seven consecutive days prior to a single dose of whole‐body X‐ray irradiation (4 Gy, 1 Gy/min). We observed that lycopene remarkably augmented sperm motility and reduced sperm abnormalities in mice following IR exposure. Histopathological analyses also revealed that lycopene ameliorated the structural damage of seminiferous tubules and enhanced the regeneration of seminiferous epithelium following IR stress. Moreover, lycopene attenuated IR‐induced oxidative stress, as evidenced by a decreasing lipid peroxidation level and an increase in the antioxidant enzyme superoxide dismutase activity. In addition, lycopene reduced the γH2AX expression and the number of TUNEL‐positive cells in the germinal epithelium, as well as restoring the imbalance of Bax/Bcl‐2 expression induced by IR exposure. Furthermore, lycopene prevented mitochondrial membrane potential depolarization and ATP reduction and preserved the activities of mitochondrial complexes I‐IV in the testes of mice after exposure to IR. Lycopene also improved mitochondrial biogenesis in testes of mice exposed to IR, presenting as restored expressions of PGC‐1α, Nrf1, and Tfam. Taken together, our results suggest that lycopene alleviates IR‐induced testicular damage, and the underlying mechanism involves at least in part the inhibition of the mitochondrial apoptotic pathway and the maintenance of mitochondrial respiration and biogenesis. The beneficial effect of lycopene highlights the therapeutic potential of this plant‐derived antioxidant against impaired spermatogenesis and male infertility induced by IR.

## Graphical abstract

1. Lycopene protects against ionizing radiation‐induced testicular damage in C57BL/6 mice.
2. The radioprotective effects of lycopene were closely related to blocking the mitochondrial apoptotic pathway and maintaining mitochondrial respiration and biogenesis.

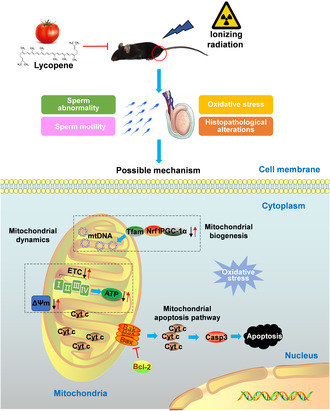

## 1 INTRODUCTION

Radiation therapy is widely used for cancer treatment, with approximately 50% of all cancer patients expected to receive it during their course of illness (Delaney et al., 2005). Despite its efficiency and cost‐effectiveness in treating cancer, radiation therapy has significantly severe adverse effects on various organs. One of the frequently documented complications of radiation therapy is male reproductive dysfunction (Velez & Ohlander, 2021). Spermatogenesis is a complex process of the formation of mature sperm cells, in which even subtle deviations can lead to sperm damage, deformity, and eventually male infertility (Toshimori et al., 2004). The rapidly dividing spermatogonial cells in spermatogenesis are appreciably susceptible to ionizing radiation (IR) (Wdowiak et al., 2019). The toxic effects of IR are primarily mediated by the generation of free radicals through water radiolysis. Excessive levels of free radicals in testicular tissue can cause damage to all biomolecules, including DNA, proteins, and lipids, resulting in the apoptosis of spermatogonial cells. Therefore, oxidative stress and spermatogonial apoptosis play critical roles in IR‐induced impairment of spermatogenesis (Asadi et al., 2021). Accordingly, agents that can suppress oxidative stress may offer protection against IR‐induced damage to spermatogenesis and reproductive function.

Although a number of chemosynthetic agents with radioprotective capability have been reported to reduce the male reproductive toxicity of IR exposure, some adverse effects, such as cutaneous adverse reactions, nausea, vomiting, and blood pressure alterations, cannot be neglected (De Felice et al., 2019; Wdowiak et al., 2019). These adverse effects have led researchers to seek out alternatives to natural ingredients. Lycopene, a natural agent with rare adverse effects reported, has recently gained much attention in radioprotective applications owing to its unique biological properties (Pirayesh Islamian & Mehrali, 2015). Lycopene is an acyclic isomer of beta‐carotene synthesized by plants and microorganisms. Its structure comprises a highly unsaturated hydrocarbon containing 11 conjugated and two unconjugated double bonds. The conjugated polyene structure is responsible for the free radical scavenging and singlet oxygen quenching activity of lycopene, making it a potent antioxidant that can provide protection against oxidative stress‐associated damage (Kim et al., 2015). Several recent studies have demonstrated the radioprotection afforded by lycopene. It is reported that lycopene supplemented by oral gavage at a dose of 5 mg/kg significantly reduced IR‐induced (A single fraction of 8 Gy at a 300 cGy/min dose rate) oxidative liver injury by reducing lipid peroxidation and increasing GSH levels and GSH‐Px and superoxide dismutase (SOD) enzyme activity in rats (Meydan et al., 2011). Coskun et al. found that lycopene administered at a dose of 6 mg/kg daily for 7 days protected against radiation‐induced esophageal toxicity in rats receiving 35 Gy Single fraction thoracic radiotherapy. (Coskun et al., 2017). Motallebnejad et al. showed that 50 mg/kg lycopene administered intraperitoneally for 10 days alleviated the severity of oral mucositis induced by gamma radiation (1400 cGy/min) in rats (Motallebnejad et al., 2020). In addition, lycopene continuously supplied in drinking water at doses of 0.15 or 0.30 mg/kg significantly reduced X‐ray radiation‐induced genetic damage in mice reticulocytes, as shown by the decreased level of micronucleus in peripheral blood and bone marrow (Dobrzyńska et al., 2019). Collectively, the radioprotective effects of lycopene in various types of tissues lead us to infer that lycopene may also antagonize the IR‐induced testicular damage.

Several lines of evidence have indicated that lycopene has a protective role in male reproduction and fertility (Durairajanayagam et al., 2014). Previous studies have demonstrated that lycopene can protect testicular damage induced by Benzo [a] pyrene (Xu, Wang, et al., 2019), cisplatin (Elsayed et al., 2022), gentamicin (Aly, 2019), and di‐(2‐ethylhexyl) phthalate (Bahrami et al., 2018) in different experimental models. One prior randomized controlled trial also revealed the protective effects of lycopene on male reproductive function. It was reported that daily supplementation with 25 mg lycopene for 12 weeks increased sperm count and sperm concentration in infertile men with oligozoospermia compared with the placebo control group (Nouri et al., 2019). In another recent clinical trial, sperm motility and morphology significantly improved in young, healthy men supplemented with 14 mg/d lactolycopene for 12 weeks compared with the placebo group (Williams et al., 2020).

Therefore, we hypothesize that lycopene probably has a protective effect on IR‐induced male reproductive system damage. Indeed, a recent experimental study observed that lycopene supplementation improved sperm quality in irradiated mice (Dobrzy Ska & Gajowik, 2020). However, the exact effects and mechanisms of spermatogenesis remain unknown. Therefore, in the present study, we aimed to investigate the effects of lycopene on IR‐induced testicular damage and what kind of mechanism may be involved in these effects of lycopene. Our results not only confirm the ameliorative effect of lycopene on radiation‐induced testicular damage but also extend previous findings by providing a possible mechanism involving inhibition of the mitochondrial apoptotic pathway and maintenance of mitochondrial respiration and biogenesis.

## 2 MATERIALS AND METHODS

### 2.1 Animals and ethics statement

Adult male C57BL/6 mice at approximately 6–8 weeks of age and weighing 22–24 g were purchased from the SPF Biotechnology Co., Ltd. (Beijing, China). All mice were kept under controlled conditions of temperature (24–26°C), relative humidity (50 ± 5%), and 12 h light‐12 h darkness rhythm. The mice were fed a standard laboratory chow and were provided with water ad libitum. The experimental procedures were authorized and approved by the local ethics and were performed in accordance with the National Research Council's Guide for the Care and Use of Laboratory Animals.

### 2.2 Experimental design

Lycopene (SL8700, Solarbio, CHN) was dissolved in corn oil and administered by oral gavage to mice. Mice were treated with vehicle (corn oil, 0.01 mL/g body weight) or lycopene at a dose of 20 mg/kg per day for seven consecutive days prior to X‐ray irradiation. The dosage and duration of lycopene administration in this study were based on previous studies (Dai et al., 2015; Ma et al., 2018). A high‐energy X‐ray irradiator (225 KV 13.2 mA, 1 Gy/Min, KUBTEC USA) was used for whole‐body irradiation. Mice were randomly divided into four groups; each group included six mice, triplicate was done to ensure accurate statistical analysis.

- Group 1 (Control group): Mice received corn oil alone.
- Group 2 (Lycopene group): Mice were administered lycopene dissolved in corn oil.
- Group 3 (IR group): Mice received corn oil for seven days, then were exposed to a single dose of whole‐body irradiation (4 Gy) with X‐rays.
- Group 4 (Lycopene + IR group): Mice were administered lycopene for seven days prior to whole‐body irradiation (4 Gy).

### 2.3 Sample collection and processing

Sperm samples were collected for sperm parameter evaluation at 4 weeks after irradiation. Mice were anesthetized with 1% pentobarbital sodium and sacrificed by cervical dislocation. Cauda epididymis tissues were dissected. Sperm suspensions were obtained as described in a previous study (Bahrami et al., 2018). Briefly, cauda epididymis tissues were incubated in 1 mL of T6 medium containing 4 mg/mL BSA for 1 h at 37°C and 5% CO_2_ in a petri dish to allow sperm to leave the epididymal tubules. All the procedures were performed at 37°C, and all equipment and reagents that came into contact with the sperm were prewarmed to and maintained at 37°C.

Testicular tissues were collected for histopathological analysis at 4 weeks after irradiation. The mice were anesthetized and sacrificed at 24 h or 4 weeks for harvesting testicular tissue samples. Testes were immediately frozen on liquid nitrogen and stored at −80°C until total protein extraction, or alternatively, fixed in Bouin's solution for histological processing.

### 2.4 Evaluation of sperm parameters

Sperm parameters were evaluated 4 weeks after irradiation. For sperm count and motility examination, samples of sperm suspensions were detected with a sperm automatic analyzer (CASA, Zeiss Lab, GER). For sperm morphology analysis, the sperm suspensions were stained with eosin dye and observed by optical microscopy (DMI8A; Leica, Germany). A total of 200 sperm in each sample were examined. Sperm abnormalities were recorded as a percentage of the total number of sperm counted.

### 2.5 Histopathological evaluation of the testis

The testes of mice were dissected out 4 weeks after irradiation, and the freshly harvested testes were immersed immediately in Bouin's solution (G1121, Servicebio, CHN) for 48 h for fixation. The samples were then dehydrated in graded ethanol, cleared in xylene, embedded in paraffin blocks, and sectioned to a thickness of 5 mm. Finally, histopathological examination was performed following hematoxylin and eosin (H&E) staining. For quantitative evaluation, the seminiferous tubule diameter and seminiferous epithelium thickness were blindly studied by a pathologist under an optical microscope using a digital imaging microscope (DMI8A, Leica, Germany). Twenty randomly selected seminiferous tubules from each section were evaluated. Histopathological damage was semiquantitatively evaluated using Johnsen's score system described in the previous study (Saad & Mahmoud, 2014). In this scoring system, all tubular sections are analyzed systematically and assigned a score from 1 to 10. Normal spermatogenesis with many spermatozoa present is assessed as having a score of 10.

### 2.6 Immunofluorescence assay

Immunofluorescence staining for expression of γ‐H2AX was conducted as described in a previous study (Xu, Xu, et al., 2019). Briefly, sections cut from the testes in different groups were incubated with 5% fetal bovine serum for 30 min at room temperature and then incubated overnight at 4°C with primary antibodies against γ‐H2AX (ab81299, Abcam, 1:200). Goat anti‐mouse Alexa Fluor 488 (A0428, Beyotime, CHN) was used for secondary staining. Nuclei were counterstained with DAPI (G1012, Servicebio, CHN). Images of the stained samples were captured using a fluorescence microscope (Olympus, Tokyo, Japan). The γH2AX expression was analyzed from the images of IF staining by using ImageJ software 1.8.0 (National Institutes of Health, United States). The data were expressed as a percentage of the fluorescence of the control group.

### 2.7 TUNEL assay

Apoptosis in the testis tissue was determined by using an apoptosis detection kit (G1507; Servicebio, CHN) following the manufacturer's instructions. Cells with intensely dark brown staining were scored as TUNEL‐positive. The number of TUNEL‐positive cells per tubule was counted with fifty random seminiferous tubules from each section.

### 2.8 Evaluation of SOD and malondialdehyde (MDA)

Mouse testis tissues were collected at 6 h after radiation and homogenized in precooled PBS. The supernatant sample was obtained by centrifugation and stored at −20°C for experiment. For the assessment of testicular MDA content, a lipid peroxidation MDA assay kit (S0131S; Beyotime, CHN) was used according to the manufacturer's instructions. For the determination of SOD, a total superoxide dismutase assay kit (S0101S; Beyotime, CHN) was used following the manufacturer's instructions.

### 2.9 Measurement of mitochondrial membrane potential (ΔΨm) and complex activities

Mitochondria were isolated by using the Tissue Mitochondria Isolation Kit (C3606; Beyotime, CHN) according to the manufacturer's instructions. All procedures were performed on ice or at 4°C. Testes were minced in mitochondrial isolation reagent A for homogenates. The homogenate was centrifuged at 600 *g* for 5 min. The supernatant fraction was then centrifuged at 11,000 *g* for 10 min to obtain the mitochondrial pellet. The supernatant was discarded, and the final pellet was resuspended in storage buffer to get a suspension of purified mitochondria, which was used for the assay of ΔΨm and Complex Activities.

According to the manufacturer's instructions for the mitochondrial membrane potential assay kit with JC‐1 (C2006, Beyotime, CHN), purified mitochondria of testicular tissue were stained with the JC‐1 staining solution at 37°C for 15 min in the dark. After being washed twice with JC‐1 staining buffer, the fluorescence value was determined by a fluorescence microplate reader (OA4209, Molecular Devices, CHN). The ratio of aggregate JC‐1 red fluorescence to Monomeric JC‐1 green fluorescence was used as an indicator to show changes in ΔΨm.

The measurements for the activities of mitochondrial respiratory chain complex I (NADH–ubiquinone oxidoreductase), complexII (succinate dehydrogenase), complex III (ubiquinol‐cytochrome c oxidoreductase), and complex IV (Cytochrome c oxidase) were carried out as described in our previous study (Qu et al., 2013). Protein concentration was determined by a BCA protein detection kit (P0012, Beyotime, CHN). The mitochondrial respiratory chain complex activities were normalized to their total protein and expressed as a ratio to control.

### 2.10 Measurement of ATP content

Based on the instructions of the ATP Assay Kit (S0026, Beyotime, CHN), testicular tissue (20 mg) was homogenized in ATP detection lysis buffer and centrifuged at 12,000 r/min for 5 min. Then, the supernatant was used to detect ATP concentration by using the Luminometer (Turner Designs, USA).

### 2.11 Western blot assay

Mouse testis tissues were collected at 24 h after radiation and lysed with RIPA lysis buffer. The protein concentrations were measured by the BCA protein detection kit. Western blotting was performed according to standard procedures. Briefly, equal amounts of the protein were separated by SDS‐PAGE, followed by transfer to polyvinylidene fluoride membranes (Bio‐Rad, USA). The membranes were incubated with antibodies against the proteins Bax (1:800, WL01637; Wanleibio, CHN), Bcl‐2 (1:500, WL01556; Wanleibio, CHN), β‐tublin (1:1000, 10094‐1‐AP; Proteintech), Tfam (1:2000, ab131607; Abcam, UK), PGC‐1α (1:1000, ab54481; Abcam, UK), and Nrf1(A5547; Abclonal, CHN) overnight at 4°C (all from Proteintech, USA). After being washed three times with TBST, the membranes were incubated with secondary antibody (HRP‐conjugated goat anti‐rabbit IgG, SA00001‐2, 1:5000; Proteintech, USA) for 1 h at room temperature. After final washes, the gray values of target bands were analyzed using Image J (version1.51; National Institutes of Health, USA) with β‐tublin as an internal control.

### 2.12 Statistical analysis

The SPSS package program (version 25 for windows) was used for statistical analysis. The data were expressed as the mean ± standard deviation (SD). Statistical significance was calculated using a one‐way analysis of variance (ANOVA) followed by the least significant difference (LSD) test. A value of *p* < .05 was considered statistically significant.

## 3 RESULTS

### 3.1 Lycopene improved sperm parameters in mice following IR exposure

**FIGURE 1:**
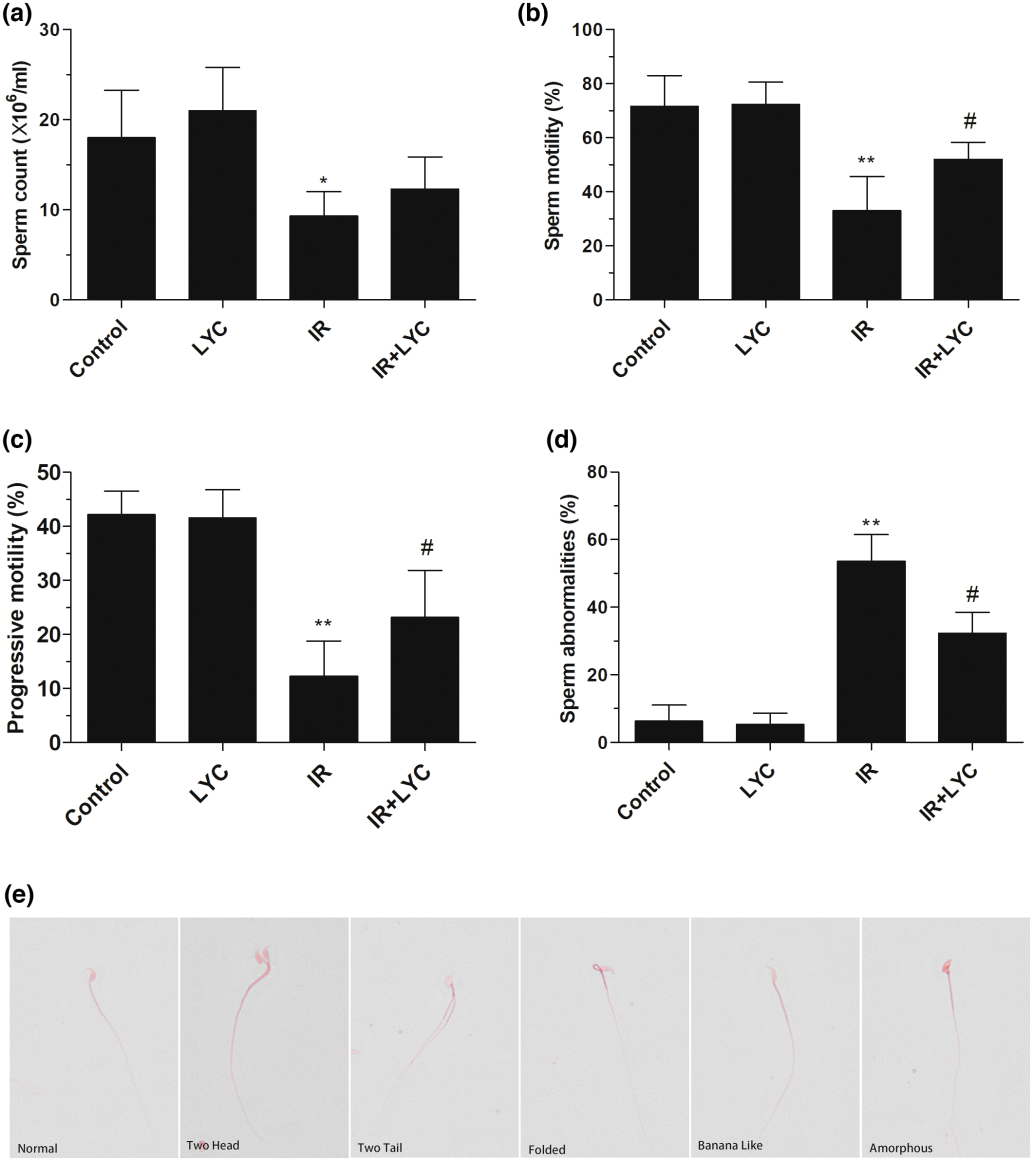
Effects of lycopene on IR‐induced sperm parameters changes. Sperm parameters, including sperm count (a) sperm motility (b) and progressive motility (c) and sperm abnormalities (d) were detected in mice 4 weeks after IR exposure with or without lycopene. Results are expressed as the mean ± SD (*n* = 6). *p < .05, **p < .01 for the comparison with the IR exposure group, ^#^ *p* < .05 for the comparison with the control group. (e) Representative images of eosin‐stained sperm after IR exposure. Sperm abnormalities were described as two‐head, two‐tail, folded, banana‐Like head, amorphous, etc. Bar = 5 μm.

Sperm parameters are associated with testicular function. Therefore, changes in the sperm parameters in the treated mice were investigated to determine the extent of the IR‐induced testicular injury and the possible protective effects of lycopene. Sperm count, motility, and abnormalities were analyzed 4 weeks after exposure to IR. As shown in Figure 1, IR exposure remarkably reduced sperm count (Figure 1a), sperm motility (Figure 1b), and progressive motility (Figure 1c), respectively, in comparison with those in the control. However, lycopene treatment significantly increased sperm motility and progressive motility compared with the IR group. The sperm count was slightly increased but no significant difference was observed in IR‐exposed mice treated with lycopene compared with IR‐exposed mice (Figure 1a). Moreover, IR exposure significantly increased the number of abnormal sperm (Figure 1d) compared with those in the control group. Meanwhile, lycopene treatment notably decreased sperm abnormalities induced by IR. Morphologically normal sperm and various sperm defects are shown in Figure 1e. Lycopene treatment alone did not appear to give rise to any significant alteration of sperm parameters compared with the control group.

### 3.2 Lycopene mitigated testicular histopathological alterations induced by IR

**FIGURE 2:**
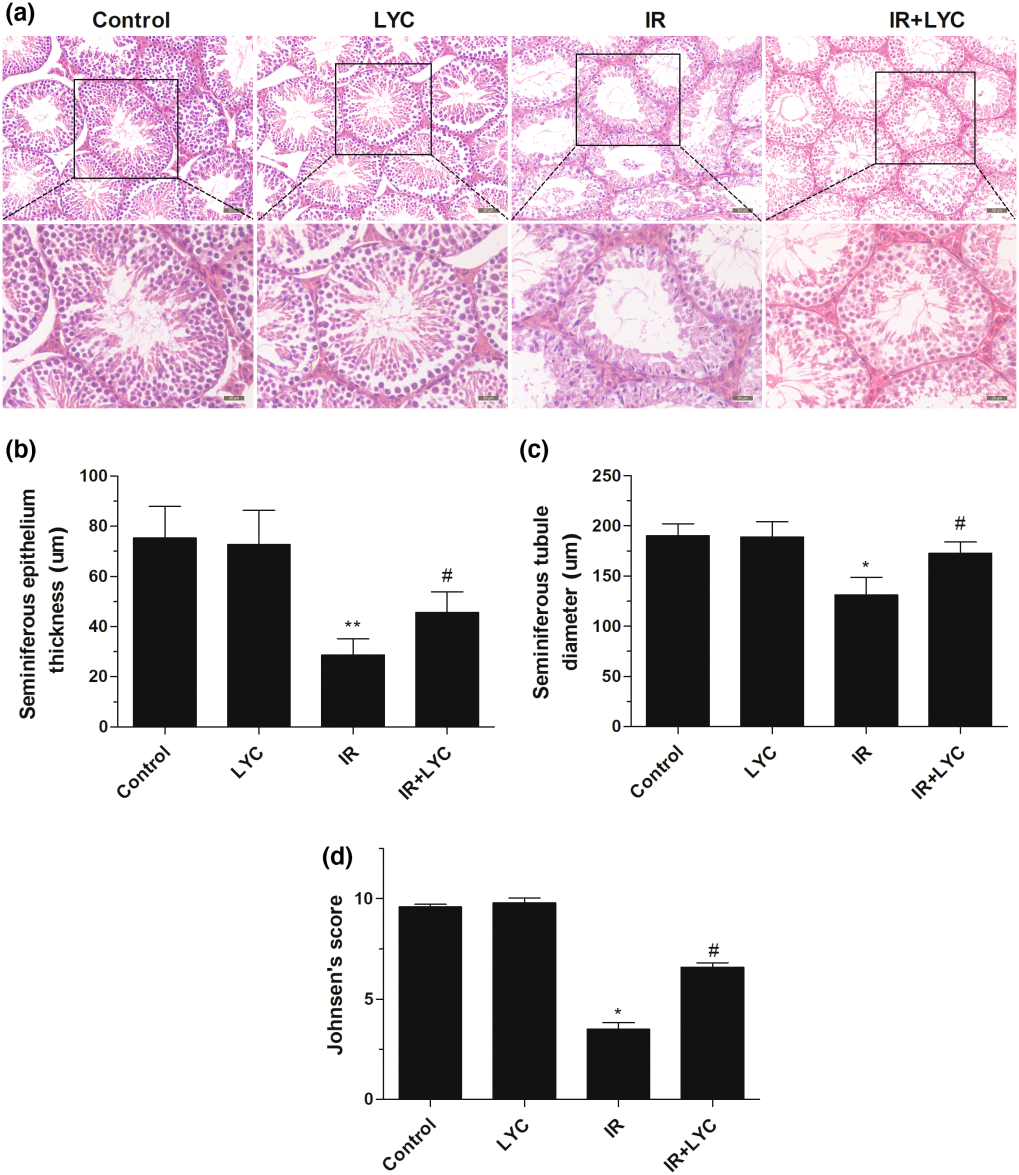
Effects of lycopene on IR‐induced testicular histopathological alterations. (a) Representative images of hematoxylin/eosin stained transverse sections of testis from different groups of mice. Scale bar: up 50 μm, down 20 μm. Quantitative evaluation of the seminiferous epithelium thickness (b) and seminiferous tubule diameter (c) from different groups of mice. (d) Johnsen's score following IR exposure and/or lycopene treatment. Results are expressed as the mean ± SD (*n* = 6). *p < .05, **p < .01 for the comparison with the IR exposure group, ^#^ *p* < .05 for the comparison with the control group.

To evaluate the effects of lycopene on testicular histopathological changes induced by IR, hematoxylin–eosin staining was performed 4 weeks after IR exposure. As shown in Figure 2a, transverse sections of testis from the control and lycopene groups exhibited normal histological features. In contrast, the IR group displayed various histopathological alterations, including atrophied seminiferous tubules disorganized spermatogenic cells in the seminiferous epithelium, and very few spermatozoa in the lumen. However, these IR‐induced histopathological changes were significantly attenuated by lycopene treatment. Morphometric analysis revealed that IR exposure caused a significant decline in epithelium thickness and seminiferous tubule diameter, respectively, compared with the control. However, lycopene treatment significantly increased the epithelium thickness and seminiferous tubule diameter, respectively, compared with the IR group (Figure 2b,c). Furthermore, the characteristics of spermatogenesis were evaluated using Johnsen's score. As depicted in Figure 2d, the IR group exhibited the lowest Johnsen's score, revealing poor spermatogenesis. However, Johnsen's score was significantly higher in the IR in combination with the lycopene group compared with the IR group. The results show the beneficial effects of lycopene on histological changes.

### 3.3 Lycopene inhibited oxidative damage in the testes of mice exposed to IR

**FIGURE 3:**
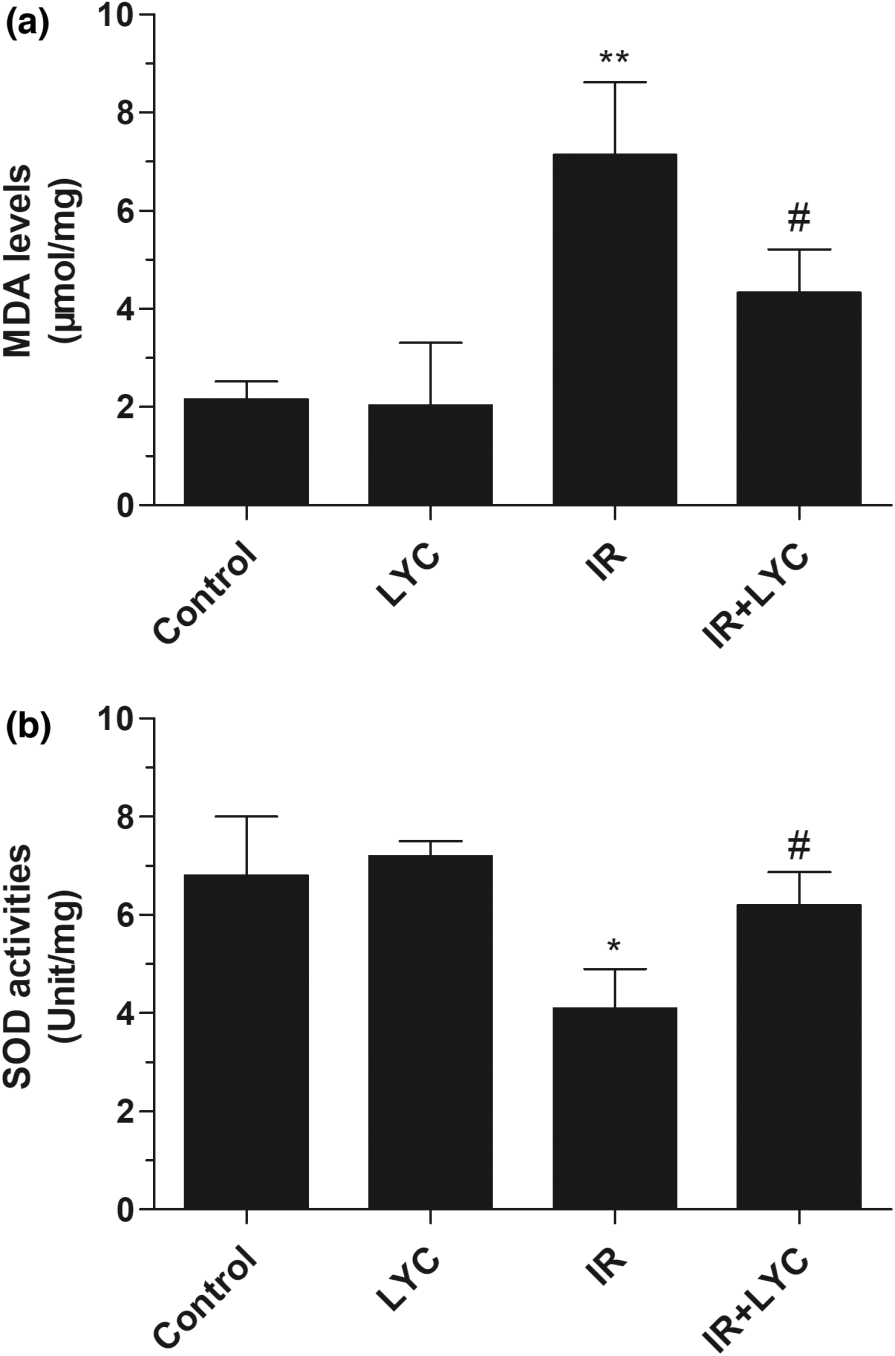
Effects of lycopene on oxidative damage in the testes of mice exposed to IR. Changes in MDA level (a) and SOD activity (b) were determined in mice 24 h after IR exposure with or without lycopene. Results are expressed as the mean ± SD (*n* = 6). *p < .05, **p < .01 for the comparison with the IR exposure group, ^#^ *p* < .05 for the comparison with the control group.

To investigate the effects of lycopene on IR‐induced oxidative damage, lipid peroxidation levels and SOD activities were detected 24 h after IR exposure. As shown in Figure 3a, MDA levels were found to significantly increase after IR exposure compared with those in the control group. As expected, lycopene treatment efficiently reduced MDA levels compared with the IR group. Lycopene‐only‐treated mice did not show any significant change in MDA levels as compared to the control group. SOD is a superoxide scavenger enzyme, and IR exposure significantly decreased SOD levels in mice compared with those in the control group, while lycopene significantly increased SOD levels in testes compared with those in the IR group, but no significant differences were observed in lycopene‐only‐treated mice (Figure 3b).

### 3.4 Lycopene inhibited DNA damage, the imbalance of Bax/Bcl‐2, and apoptosis in the testes of mice exposed to IR

**FIGURE 4:**
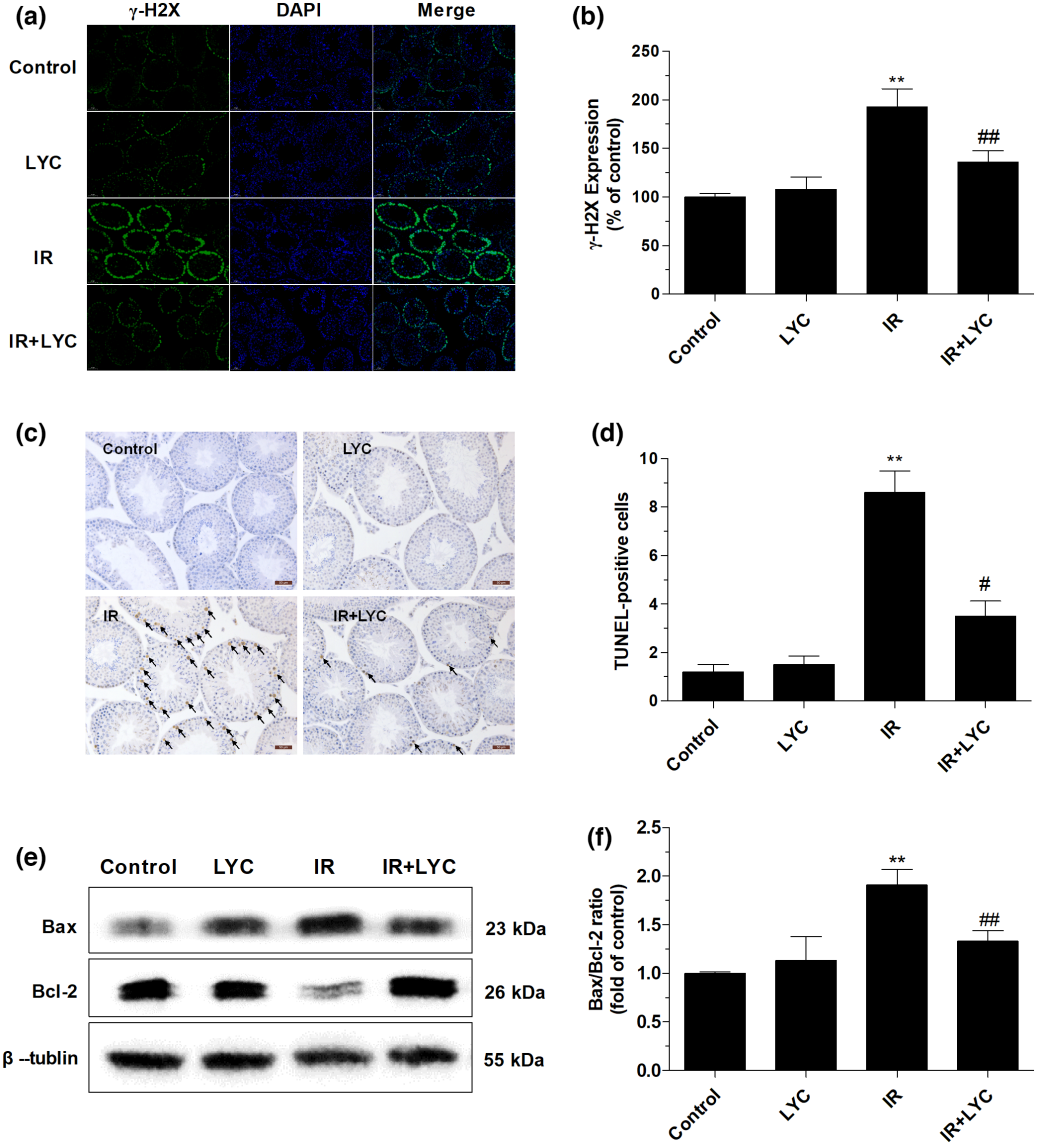
Effects of lycopene on DNA damage and apoptosis in the testes of mice exposed to IR. (a) Representative immunofluorescence images of γH2AX expression in testicular sections from different groups of mice at 24 h after IR exposure. Scale bar: 50 μm. (b) Quantitative analysis of γ‐H2AX expression. (c) Representative images of TUNEL‐stained transverse sections of testis from different groups of mice at 24 h after IR exposure. The black arrow indicates apoptotic cells, which have brown staining within their nuclei. The apoptotic cells are primarily located in the outer layer of the seminiferous tubules. Scale bar: 50 μm. (d) The number of TUNEL‐positive cells per seminiferous tubule in the testis sections of different groups of mice. (e) Representative Western blot images of Bax and Bcl‐2 protein expression in testis from different groups at 24 h after IR exposure. (f) The bar graph represents the densitometric quantification of the Bax/Bcl‐2 ratio. β‐tublin was used as a protein loading control. Results are expressed as the mean ± SD (*n* = 6). **p < .01 for the comparison with the IR exposure group, ^#^ *p* < .05, ^##^ *p* < .01 for the comparison with the control group.

It is well accepted that the formation of DNA strand breaks and subsequent induction of apoptosis are potential mechanisms of IR testicular damage. Therefore, γH2AX expression as a marker of DNA double‐strand break formation was determined by immunofluorescence assay at 24 h post‐IR in different groups of mice. As shown in Figure 4a,b, γH2AX expression was markedly higher in the IR group compared with the control group, indicating IR caused severe DNA damage, while lycopene administration significantly inhibited IR‐induced DNA damage compared with the IR group.

To determine the effects of lycopene on IR‐induced apoptosis, the TUNEL assay was performed at 24 h post‐IR in each group. As shown in Figure 4c,d, TUNEL‐positive cells were rarely observed in both the control and lycopene‐only treated groups. In contrast, a significantly increased number of TUNEL‐positive cells were detected in the outer layer of the seminiferous tubules in the IR exposure group as compared with the control group, which were primarily spermatogonial cells and primary spermatocytes. However, lycopene largely reduced the number of apoptotic cells as compared with the IR group, indicating that the protective effects of lycopene on IR‐induced testicular damage might be mediated by its anti‐apoptotic activity.

To investigate the mechanism underlying the protection of lycopene against IR‐induced apoptosis in testes, the expression of Bcl‐2 family members (Bax, Bcl‐2) was determined using western blot analysis at 24 h post‐IR in each group. As shown in Figure 4e,f, IR exposure caused a significant upregulation of Bax expression and a significant reduction in Bcl‐2, leading to an elevated Bax/Bcl‐2 ratio as compared to the control group. However, the IR exposure‐induced increase in Bax/Bcl‐2 ratio was significantly reversed by lycopene treatment, suggesting that lycopene plays a role in maintaining the balance between anti‐apoptotic and pro‐apoptotic protein expression. The results indicated that the mitochondrial apoptosis pathway was activated by IR exposure, while lycopene treatment might potentially counteract this pathway.

### 3.5 Lycopene prevented IR‐induced mitochondrial respiratory dysfunction in mice following IR exposure

**FIGURE 5:**
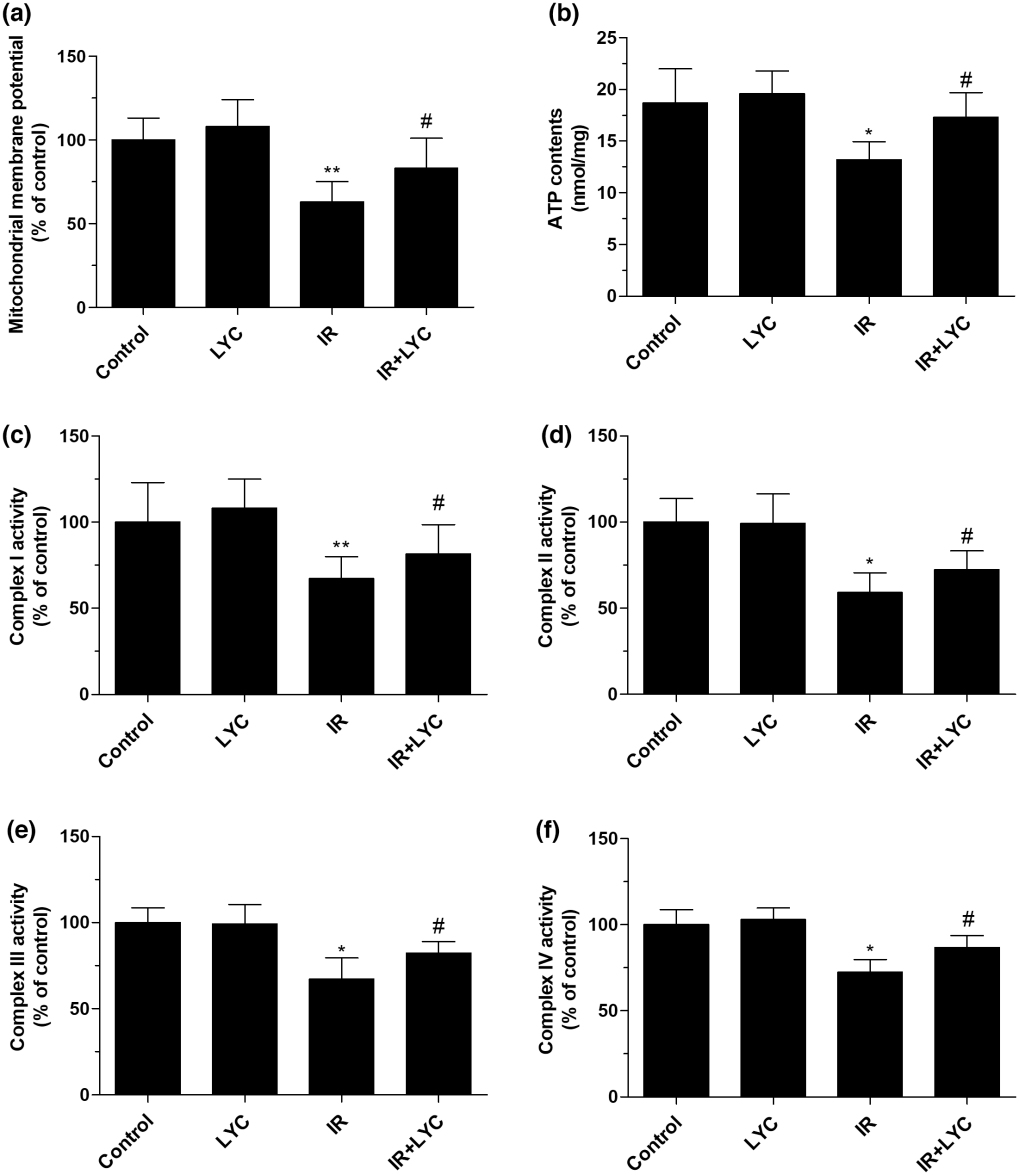
Effects of lycopene on the reduced ΔΨM, ATP contents, and mitochondrial complexes I‐IV activities in the testes of mice exposed to IR. Changes in ΔΨM (a) ATP contents (b) and mitochondrial complex I–IV activities (c–f) were detected in mice 24 h after IR exposure with or without lycopene. Results are expressed as the mean ± SD (*n* = 6). *p < .05, **p < .01 for the comparison with the IR exposure group, and ^#^ *p* < .05 for the comparison with the control group.

To explore whether the protective effects of lycopene are relevant to preserving mitochondrial respiratory function, the ΔΨM, ATP contents, and mitochondrial complex I‐IV activities were evaluated at 24 h post‐IR in different groups of mice. It was observed that IR exposure significantly reduced ΔΨM compared with the control, but lycopene treatment significantly reversed the IR‐induced ΔΨM depolarization compared with the IR group. (Figure 5a). Subsequently, it was found that ATP content was significantly reduced from 18.7 nmol/mg protein in the control group to 13.2 nmol/mg protein in the IR group. However, the IR‐induced reduction in ATP concentration was markedly prevented by lycopene pretreatment (Figure 5b). As shown in Figure 5c–f, IR exposure caused significant decreases in the activities of mitochondrial respiratory chain complexes I‐IV respectively, in comparison with those in the control; however, these decreases were strikingly inhibited by lycopene pretreatment as compared with the IR group.

### 3.6 Lycopene improved mitochondrial biogenesis in the testes of mice exposed to IR

**FIGURE 6:**
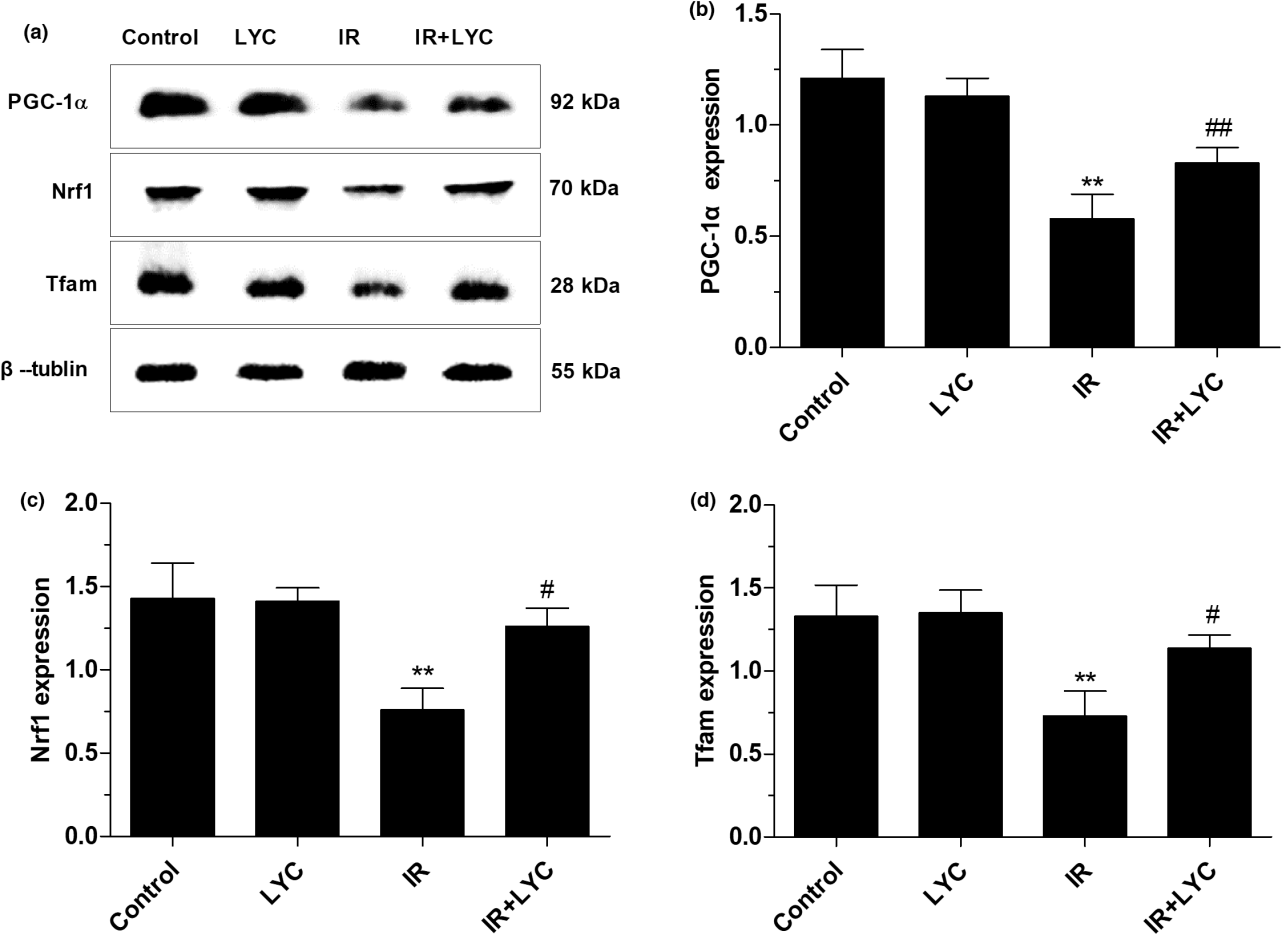
Effects of lycopene on the decreased PGC‐1α, Nrf1, and Tfam expression in the testes of mice exposed to IR. (a) Representative Western blot images of PGC‐1α, Nrf1, and Tfam protein expression in testis from different groups at 24 h after IR exposure. The bar graph represents densitometric quantification of PGC‐1α (b) Nrf1 (c) and Tfam (d) β‐tublin was used as a protein loading control. Results are expressed as the mean ± SD *n* = 6 for each group. **p < .01 for the comparison with the IR exposure group, ^#^ *p* < .05, and ^##^ *p* < .01 for the comparison with the control group.

To investigate whether lycopene‐mediated radioprotection is related to improved mitochondrial biogenesis, the expression of the mitochondrial biogenesis‐related proteins PGC‐1α, Nrf1, and Tfam was detected by Western blot at 24 h post‐IR in different groups of mice. As shown in Figure 6, the protein expressions of PGC‐1α, Nrf1, and Tfam were significantly decreased in the IR groups compared with the control groups, respectively. However, these IR‐induced reductions in PGC‐1α, Nrf1, and Tfam expression were efficiently prevented by lycopene compared with the IR groups, respectively.

## 4 DISCUSSION

**FIGURE 7:**
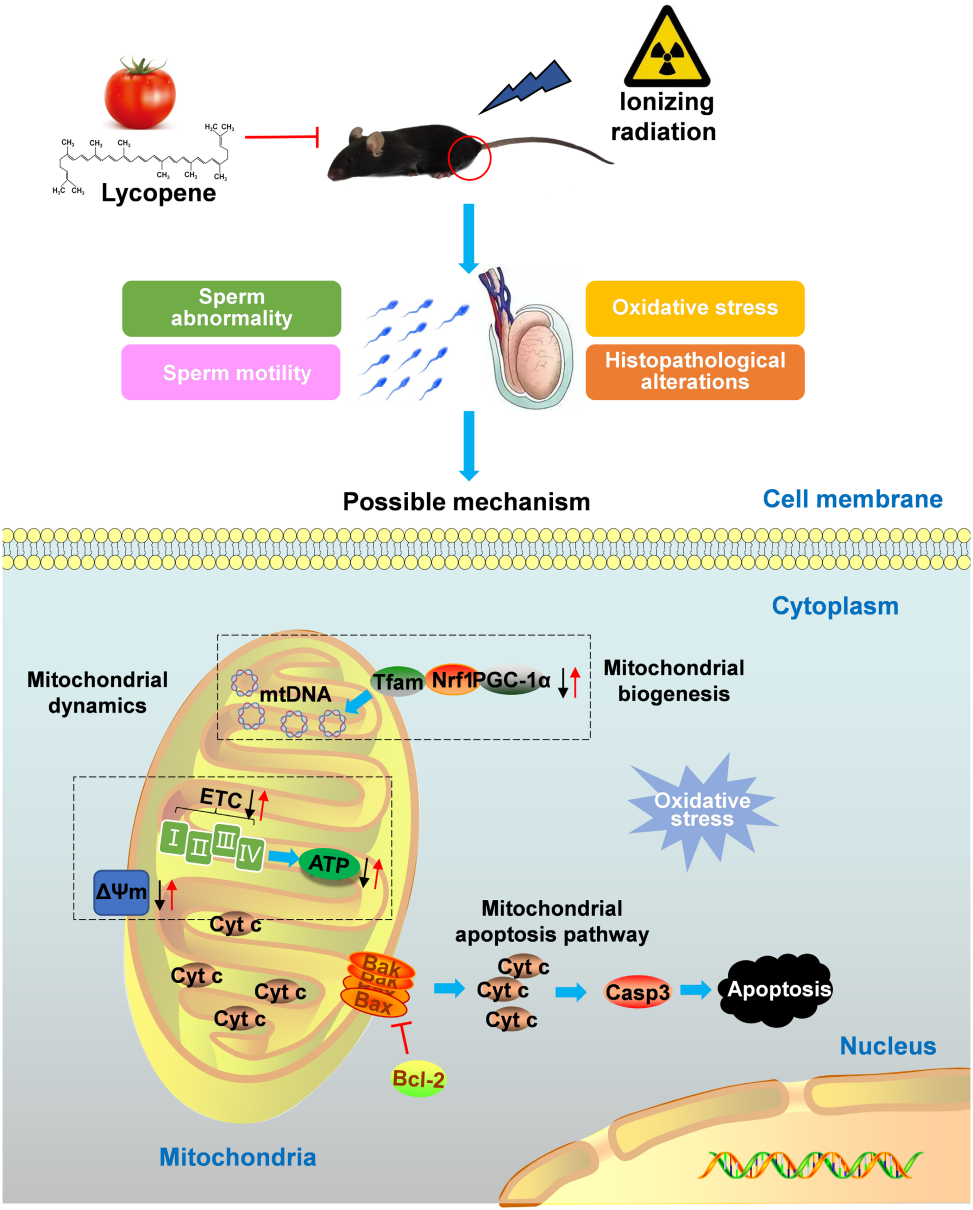
Proposed mechanisms underlying the radioprotective effect of lycopene in mice testes. Lycopene protects against ionizing radiation‐induced testicular damage in mice. The possible mechanism is involved in blocking the mitochondrial apoptotic pathway, inhibiting mitochondrial respiratory dysfunction, and improving mitochondrial biogenesis.

Following the increasing application of IR in medicine, including diagnosis and treatment, the number of reports on male reproductive dysfunction has mounted steadily (Qu et al., 2019). In recent decades, several therapeutic strategies have been explored to suppress testicular damage and counteract male infertility related to irradiation, but their effectiveness is limited (Jiang et al., 2013; Samarth & Samarth, 2009; Silva et al., 2016). Lycopene, which belongs to the carotenoid family, seems to be a promising potential treatment option for IR‐induced male infertility due to its potent antioxidant and antiapoptotic properties (Antonuccio et al., 2020). In the present study, we found that lycopene treatment preserved the sperm characteristics and maintained the spermatogenesis in a mouse model of IR‐induced testicular toxicity by blocking mitochondrial apoptotic pathways, inhibiting mitochondrial respiratory dysfunction, and improving mitochondrial biogenesis (Figure 7).

Testicular spermatogenesis is a delicately regulated process that is extremely sensitive to IR (Meistrich, 2013). Sperm are produced by the process of spermatogenesis within the seminiferous tubules of the testis. Sperm parameters directly indicate male reproductive health. Sperm count, motility, and abnormal morphology are crucial factors affecting fertility and are commonly used parameters for assessing male infertility (Nallella et al., 2006). A number of studies have demonstrated that IR exposure can adversely affect sperm parameters, leading to fertility impairment (Kesari et al., 2018). Therefore, we first aimed to determine the potential protective effects of lycopene on testicular spermatogenesis through sperm parameter evaluations. Here, we found that the addition of lycopene increased sperm motility and reduced the number of abnormal sperm. Our results confirm a previous study that found that lycopene administration increased the sperm count and reduced the percentage of morphologically abnormal spermatozoa in mice after 0.5 or 1 Gy doses of irradiation (Dobrzy Ska & Gajowik, 2020). However, our results indicated that there was no significant effect of lycopene on sperm count in our mouse model. In the study of Dobrzy Ska et al., lycopene administration (0.15 and 0.30 mg/kg) in the drinking water was started at 24 h or on Day 8 after irradiation and lasted for 2 weeks. In contrast, the mice were administered lycopene for 7 days before, rather than after, irradiation in our study. Additionally, the irradiation dose and administration dosage of lycopene in the two studies were different. The differences in experimental design between our study and the study by Dobrzy Ska et al mentioned above may be important reasons behind the observed differences. Disturbances in spermatogenesis give rise to sperm abnormalities. (Tuttelmann et al., 2018), we therefore further investigated the effects of lycopene on spermatogenesis by evaluating histopathological changes in mice testis. In line with previous studies (Aly, 2019), we observed that lycopene reduced seminiferous tubule damage, as shown by the increased mean tubule diameter and epithelium thickness, and improved regeneration of seminiferous epithelium, as shown by the increased Johnsen's score. This indicated that lycopene protected the IR‐impaired spermatogenesis in mice. Together, these results extend the range of protective applications of lycopene for male reproductive function.

Possible mechanisms underlying such a beneficial effect of lycopene may be its ability to counter oxidative stress and reduce the degree of apoptosis, as demonstrated in previous studies (Meng et al., 2022; Palabiyik et al., 2013; Tian et al., 2018). The involvement of oxidative stress in IR‐induced spermatogenesis damage has been well established (Turner & Lysiak, 2008). MDA is a by‐product of lipid peroxidation and is widely used as a reliable marker of tissue oxidative stress. Additionally, SOD is a superoxide scavenger enzyme belonging to the endogenous antioxidant defense system and is recognized as an indicator of antioxidative capacity. Therefore, we measured the MDA content and SOD activity to explore the possible mechanisms of the protective effects of lycopene observed in our study. We found that lycopene treatment protected mice against IR‐induced oxidative stress, as shown by the suppressed MDA levels and the restored SOD activities. These findings indicated that the radioprotective ability of lycopene was strongly related to its antioxidant property. Our results are consistent with previous studies that found that lycopene protected testicular oxidative damage caused by torsion/detorsion through increasing SOD activity and reducing lipid peroxidation levels (Guzel et al., 2016).

Apoptosis plays a crucial role in maintaining efficient spermatogenesis by managing and eliminating defective germ cells from the seminiferous epithelium. Oxidative stress induction by radiation causes DNA damage and triggers an apoptotic cascade, which is the major mechanism of radiation‐induced cell death. The phosphorylated H2AX (γH2AX) is a specific mediator of the cellular response to DNA damage (Li et al., 2006) and is regarded as a specific, sensitive biomarker of DNA damage induced by various chemicals/stress factors (Redon et al., 2002). Here, we evaluate IR‐induced DNA damage by detecting γH2AX formation. A higher γH2AX expression and more TUNEL‐positive cells were found in the testicular tissues of irradiated mice compared with those of controls, indicating IR‐caused DNA damage and apoptosis. Multiple lines of evidence indicate that the mitochondria‐dependent intrinsic pathway is the main form of IR‐induced apoptosis (Cao et al., 2019). The Bcl family plays a significant role in the regulation of mitochondrial apoptosis (Brunelle & Letai, 2009). Among these, Bax induces apoptosis by acting on mitochondria and regulating caspase activity, while Bcl‐2 counters the action of Bax and consequently inhibits apoptosis. Therefore, the Bax/Bcl‐2 protein ratio is of great significance for determining survival or death following apoptotic stimulation. Western blot results demonstrated that IR exposure strikingly increased the pro‐apoptotic Bax expression and significantly decreased the anti‐apoptotic Bcl‐2 expression, resulting in an increased Bax/Bcl‐2 protein ratio. It is suggested that the mitochondria‐dependent apoptotic process was strongly stimulated by IR. Interestingly, lycopene treatment not only significantly decreased the ratio of Bax/Bcl‐2 expression but also reduced the γH2AX expression and the number of TUNEL‐positive cells in testicular tissues, demonstrating that lycopene remarkably modulated and reduced mitochondria‐dependent apoptotic processes. These results are in line with previous studies that reported the anti‐apoptotic effects of lycopene on testicular damage (Türk et al., 2010; Xu, Wang, et al., 2019). Taken together, we believe that lycopene could exert a radiation protection effect on the testis by reducing DNA damage‐initiated apoptosis.

Mitochondria play a central role in the regulation and control of apoptosis in the testis, and their damage is closely related to male infertility. Several studies found IR exposure can impair mitochondria, leading to membrane structure damage and then ΔΨM depolarization (Said et al., 2019; Szumiel, 2015). Stable ΔΨM is the basis for maintaining mitochondrial respiratory chain enzyme complex activity and ATP synthesis, while ΔΨM loss causes mitochondrial respiratory dysfunction and is recognized as an early signal of apoptosis (Birch‐Machin & Turnbull, 2001). In the present study, we found that lycopene attenuated IR‐induced ΔΨM loss, ATP reduction, and decreases in the activities of mitochondrial complexes I‐IV. These results implied the potential of lycopene in maintaining mitochondrial respiratory function. Furthermore, mitochondrial biogenesis is a key element for mitochondrial oxidative phosphorylation and ATP synthesis. PGC‐1α is a nodal regulator of mitochondrial biogenesis and respiration, and its downstream target Nrf1 is associated with mediating the expression of several transcription factors. Nrf1 not only participates in the regulation of nuclear genes encoding subunits of the mitochondrial respiratory complexes but also regulates the transcription of mitochondrial transcription factor A (Tfam) (Fernandez‐Marcos & Auwerx, 2011). Tfam plays multiple roles in mitochondrial DNA (mtDNA) stabilization and mtDNA transcription (Kang & Hamasaki, 2005). Previous studies have shown that PGC‐1α, Nrf1, and Tfam are involved in the pathological processes of mitochondrial damage in the testes of mice exposed to Aflatoxin B1, indicating the functional role of these regulators in mitochondrial biogenesis (Huang et al., 2020). Our results are consistent with previous studies that found that IR exposure caused significant reductions in PGC‐1α, Nrf1, and Tfam expressions. However, lycopene effectively restored the expression of these regulators, suggesting lycopene prevented the testicular mitochondrial biogenesis disturbed by IR. Previous studies have shown that mitochondrial protection is the mechanism underlying the male reproductive protective effects of lycopene (Aly et al., 2012; Boeira et al., 2014). Our research expands on previous findings that the maintenance of mitochondrial function by lycopene not only involves preserving mitochondrial respiratory function and energy metabolism, as evidenced by inhibited ΔΨM depolarization, improved activities of mitochondrial complexes, and ATP generation, but also maintains mitochondrial biogenesis by regulating PGC‐1α, Nrf1, and Tfam. These results provide new clues for a deeper understanding of the protective mechanism of lycopene.

The limitation of the present study is the use of a single high‐dose IR to identify acute testicular damage histopathologically. Although it is difficult to interpret a single‐fraction 4 Gy IR dose into a fractionated radiation therapy regimen, the findings in this mouse model study are still encouraging. However, these findings should only be regarded as a preclinical basis for further clinical studies rather than a recommendation for clinical use for the treatment of male reproductive function injuries induced by IR.

## 5 CONCLUSIONS

In this study, we demonstrated that lycopene protected against IR‐induced testicular damage in C57BL/6 mice. The mechanism underlying the radioprotective effects of lycopene involves blocking the mitochondrial apoptotic pathway and maintaining mitochondrial respiration and biogenesis. Our research suggests that lycopene has a beneficial effect on IR‐related male reproductive damage, offering a new perspective on this compound for combating radiation therapy‐related male infertility complications. However, further studies are still needed to confirm these findings obtained in the mouse model.

## AUTHOR CONTRIBUTIONS

**Mingyue Qu:** Data curation (equal); formal analysis (equal); investigation (equal); methodology (equal); software (equal); visualization (equal); writing – original draft (equal). **Qican He:** Data curation (equal); formal analysis (equal); investigation (equal); methodology (equal); software (equal); visualization (equal); writing – original draft (equal). **Baoshi Guo:** Conceptualization (lead); project administration (lead); resources (lead); supervision (lead); validation (lead); writing – review and editing (lead).

## FUNDING INFORMATION

This research did not receive any specific grants from funding agencies in the public, commercial, or not‐for‐profit sectors.

## CONFLICT OF INTEREST STATEMENT

The authors declare that there is no financial or non‐financial conflict of interest in the study.

## ETHICS STATEMENT

All the authors have approved that the submitted works are original, and the paper has not been published and is not being considered for publication elsewhere.

## Data Availability

The data that support the findings of this study are available on request from the corresponding author. The data are not publicly available due to privacy or ethical restrictions.

## References

[fsn33794-bib-0001] Aly, H. (2019). Testicular toxicity of gentamicin in adult rats: Ameliorative effect of lycopene. Human & Experimental Toxicology, 38(11), 1302–1313. 10.1177/0960327119864160 31319718

[fsn33794-bib-0002] Aly, H. A. , El‐Beshbishy, H. A. , & Banjar, Z. M. (2012). Mitochondrial dysfunction induced impairment of spermatogenesis in LPS‐treated rats: Modulatory role of lycopene. European Journal of Pharmacology, 677(1–3), 31–38. 10.1016/j.ejphar.2011.12.027 22222822

[fsn33794-bib-0003] Antonuccio, P. , Micali, A. , Puzzolo, D. , Romeo, C. , Vermiglio, G. , Squadrito, V. , Freni, J. , Pallio, G. , Trichilo, V. , Righi, M. , Irrera, N. , Altavilla, D. , Squadrito, F. , Marini, H. R. , & Minutoli, L. (2020). Nutraceutical effects of lycopene in experimental varicocele: An “in vivo” model to study male infertility. Nutrients, 12(5), 1536–1537. 10.3390/nu12051536 32466161 PMC7284888

[fsn33794-bib-0004] Asadi, A. , Ghahremani, R. , Abdolmaleki, A. , & Rajaei, F. (2021). Role of sperm apoptosis and oxidative stress in male infertility: A narrative review. International Journal of Reproductive Biomedicine, 19(6), 493–504. 10.18502/ijrm.v19i6.9371 34401644 PMC8350854

[fsn33794-bib-0005] Bahrami, N. , Mehrzadi, S. , Goudarzi, M. , Mansouri, E. , & Fatemi, I. (2018). Lycopene abrogates di‐(2‐ethylhexyl) phthalate induced testicular injury by modulating oxidative, endocrine and inflammatory changes in mice. Life Sciences, 207, 265–271. 10.1016/j.lfs.2018.06.009 29886059

[fsn33794-bib-0006] Birch‐Machin, M. A. , & Turnbull, D. M. J. M. C. B. (2001). Assaying mitochondrial respiratory complex activity in mitochondria isolated from human cells and tissues. Methods in Cell Biology, 65(65), 97–117. 11381612 10.1016/s0091-679x(01)65006-4

[fsn33794-bib-0007] Boeira, S. P. , Filho, C. B. , Del'Fabbro, L. , Roman, S. S. , Royes, L. F. , Fighera, M. R. , Jessé, C. R. , Oliveira, M. S. , & Furian, A. F. (2014). Lycopene treatment prevents hematological, reproductive and histopathological damage induced by acute zearalenone administration in male Swiss mice. Experimental and Toxicologic Pathology, 66(4), 179–185. 10.1016/j.etp.2014.01.002 24503513

[fsn33794-bib-0008] Brunelle, J. K. , & Letai, A. (2009). Control of mitochondrial apoptosis by the Bcl‐2 family. Journal of Cell Science, 122(Pt 4), 437–441. 10.1242/jcs.031682 19193868 PMC2714431

[fsn33794-bib-0009] Cao, X. , Wen, P. , Fu, Y. , Gao, Y. , Qi, X. , Chen, B. , Tao, Y. , Wu, L. , Xu, A. , Lu, H. , & Zhao, G. (2019). Radiation induces apoptosis primarily through the intrinsic pathway in mammalian cells. Cellular Signalling, 62, 109337. 10.1016/j.cellsig.2019.06.002 31173879

[fsn33794-bib-0010] Coskun, H. , Andic, F. , Daglıoglu, Y. K. , Doran, F. , Sahin, K. , Tunalı, C. , & Kucuk, O. (2017). Lycopene in the prevention of radiation‐induced esophagitis. Nutrition and Cancer, 69(2), 319–329. 10.1080/01635581.2017.1265133 28094572

[fsn33794-bib-0011] Dai, C. , Tang, S. , Deng, S. , Zhang, S. , Zhou, Y. , Velkov, T. , Li, J. , & Xiao, X. (2015). Lycopene attenuates colistin‐induced nephrotoxicity in mice via activation of the Nrf2/HO‐1 pathway. Antimicrobial Agents and Chemotherapy, 59(1), 579–585. 10.1128/AAC.03925-14 25385104 PMC4291401

[fsn33794-bib-0012] De Felice, F. , Marchetti, C. , Marampon, F. , Cascialli, G. , Muzii, L. , & Tombolini, V. (2019). Radiation effects on male fertility. Andrology, 7(1), 2–7. 10.1111/andr.12562 30411532

[fsn33794-bib-0013] Delaney, G. , Jacob, S. , Featherstone, C. , & Barton, M. (2005). The role of radiotherapy in cancer treatment: Estimating optimal utilization from a review of evidence‐based clinical guidelines. Cancer, 104(6), 1129–1137. 10.1002/cncr.21324 16080176

[fsn33794-bib-0014] Dobrzy Ska, M. G. M. , & Gajowik, A. (2020). Amelioration of sperm count and sperm quality by lycopene supplementation in irradiated mice. Reproduction, Fertility, and Development, 32(12), 1040–1047. 10.1071/rd19433 32731920

[fsn33794-bib-0015] Dobrzyńska, M. M. , Gajowik, A. , & Radzikowska, J. (2019). The effect of lycopene supplementation on radiation‐induced micronuclei in mice reticulocytes in vivo. Radiation and Environmental Biophysics, 58(3), 425–432. 10.1007/s00411-019-00795-0 31123854 PMC6609584

[fsn33794-bib-0016] Durairajanayagam, D. , Agarwal, A. , Ong, C. , & Prashast, P. (2014). Lycopene and male infertility. Asian Journal of Andrology, 16(3), 420–425. 10.4103/1008-682X.126384 24675655 PMC4023371

[fsn33794-bib-0017] Elsayed, A. , Elkomy, A. , Alkafafy, M. , Elkammar, R. , El‐Shafey, A. , Soliman, A. , & Aboubakr, M. (2022). Testicular toxicity of cisplatin in rats: Ameliorative effect of lycopene and N‐acetylcysteine. Environmental Science and Pollution Research International, 29(16), 24077–24084. 10.1007/s11356-021-17736-4 34825328

[fsn33794-bib-0018] Fernandez‐Marcos, P. J. , & Auwerx, J. (2011). Regulation of PGC‐1α, a nodal regulator of mitochondrial biogenesis. The American Journal of Clinical Nutrition, 93(4), 884S–890S. 21289221 10.3945/ajcn.110.001917 PMC3057551

[fsn33794-bib-0019] Guzel, M. , Sonmez, M. F. , Bastug, O. , Aras, N. F. , Ozturk, A. B. , Kucukaydin, M. , & Turan, C. (2016). Effectiveness of lycopene on experimental testicular torsion. Journal of Pediatric Surgery, 51(7), 1187–1191. 10.1016/j.jpedsurg.2015.11.008 26703432

[fsn33794-bib-0020] Huang, W. , Cao, Z. , Yao, Q. , Ji, Q. , Zhang, J. , & Li, Y. (2020). Mitochondrial damage are involved in Aflatoxin B(1)‐induced testicular damage and spermatogenesis disorder in mice. Sci Total Environ, 701, 135077. 10.1016/j.scitotenv.2019.135077 31733399

[fsn33794-bib-0021] Jiang, Z. , Xu, B. , Yang, M. , Li, Z. , Zhang, Y. , & Jiang, D. (2013). Protection by hydrogen against gamma ray‐induced testicular damage in rats. Basic & Clinical Pharmacology & Toxicology, 112(3), 186–191. 10.1111/bcpt.12016 22998562

[fsn33794-bib-0022] Kang, D. , & Hamasaki, N. (2005). Mitochondrial transcription factor a in the maintenance of mitochondrial DNA: Overview of its multiple roles. Annals of the New York Academy of Sciences, 1042, 101–108. 15965051 10.1196/annals.1338.010

[fsn33794-bib-0023] Kesari, K. K. , Agarwal, A. , & Henkel, R. (2018). Radiations and male fertility. Reproductive Biology and Endocrinology, 16(1), 118. 10.1186/s12958-018-0431-1 30445985 PMC6240172

[fsn33794-bib-0024] Kim, J. Y. , Lee, J. S. , Han, Y. S. , Lee, J. H. , Bae, I. , Yoon, Y. M. , Kwon, S. M. , & Lee, S. H. (2015). Pretreatment with lycopene attenuates oxidative stress‐induced apoptosis in human mesenchymal stem cells. Biomol Ther (Seoul), 23(6), 517–524. 10.4062/biomolther.2015.085 26535076 PMC4624067

[fsn33794-bib-0025] Li, Z. , Yang, J. , & Huang, H. (2006). Oxidative stress induces H2AX phosphorylation in human spermatozoa. FEBS Letters, 580(26), 6161–6168. 10.1016/j.febslet.2006.10.016 17064697

[fsn33794-bib-0026] Ma, S. , Li, R. , Gong, X. , Shi, W. , & Zhong, X. (2018). Lycopene reduces in utero bisphenol A exposure‐induced mortality, benefits hormones, and development of reproductive organs in offspring mice. Environmental Science and Pollution Research International, 25(24), 24041–24051. 10.1007/s11356-018-2395-2 29948678

[fsn33794-bib-0027] Meistrich, M. L. (2013). Effects of chemotherapy and radiotherapy on spermatogenesis in humans. Fertility and Sterility, 100(5), 1180–1186. 10.1016/j.fertnstert.2013.08.010 24012199 PMC3826884

[fsn33794-bib-0028] Meng, X. , Li, L. , An, H. , Deng, Y. , Ling, C. , Lu, T. , Song, G. , & Wang, Y. (2022). Lycopene alleviates titanium dioxide nanoparticle‐induced testicular toxicity by inhibiting oxidative stress and apoptosis in mice. Biological Trace Element Research, 200(6), 2825–2837. 10.1007/s12011-021-02881-1 34396458

[fsn33794-bib-0029] Meydan, D. , Gursel, B. , Bilgici, B. , Can, B. , & Ozbek, N. (2011). Protective effect of lycopene against radiation‐induced hepatic toxicity in rats. The Journal of International Medical Research, 39(4), 1239–1252. 10.1177/147323001103900412 21986126

[fsn33794-bib-0030] Motallebnejad, M. , Zahedpasha, S. , Moghadamnia, A. A. , Kazemi, S. , Moslemi, D. , Pouramir, M. , & Asgharpour, F. (2020). Protective effect of lycopene on oral mucositis and antioxidant capacity of blood plasma in the rat exposed to gamma radiation. Caspian Journal of Internal Medicine, 11(4), 419–425. 10.22088/cjim.11.4.419 33680384 PMC7911765

[fsn33794-bib-0031] Nallella, K. P. , Sharma, R. K. , Aziz, N. , & Agarwal, A. (2006). Significance of sperm characteristics in the evaluation of male infertility. Fertility and Sterility, 85(3), 629–634. 10.1016/j.fertnstert.2005.08.024 16500330

[fsn33794-bib-0032] Nouri, M. , Amani, R. , Nasr‐Esfahani, M. , & Tarrahi, M. J. (2019). The effects of lycopene supplement on the spermatogram and seminal oxidative stress in infertile men: A randomized, double‐blind, placebo‐controlled clinical trial. Phytotherapy Research, 33(12), 3203–3211. 10.1002/ptr.6493 31468596

[fsn33794-bib-0033] Palabiyik, S. S. , Erkekoglu, P. , Zeybek, N. D. , Kizilgun, M. , Baydar, D. E. , Sahin, G. , & Giray, B. K. (2013). Protective effect of lycopene against ochratoxin A induced renal oxidative stress and apoptosis in rats. Experimental and Toxicologic Pathology, 65(6), 853–861. 10.1016/j.etp.2012.12.004 23332503

[fsn33794-bib-0034] Pirayesh Islamian, J. , & Mehrali, H. (2015). Lycopene as a carotenoid provides radioprotectant and antioxidant effects by quenching radiation‐induced free radical singlet oxygen: An overview. Cell Journal, 16(4), 386–391. 10.22074/cellj.2015.485 25685729 PMC4297477

[fsn33794-bib-0035] Qu, M. , Nan, X. , Gao, Z. , Guo, B. , Liu, B. , & Chen, Z. (2013). Protective effects of lycopene against methylmercury‐induced neurotoxicity in cultured rat cerebellar granule neurons. Brain Research, 1540, 92–102. 10.1016/j.brainres.2013.10.005 24120987

[fsn33794-bib-0036] Qu, N. , Itoh, M. , & Sakabe, K. (2019). Effects of chemotherapy and radiotherapy on spermatogenesis: The role of testicular immunology. International Journal of Molecular Sciences, 20(4), 957–958. 10.3390/ijms20040957 30813253 PMC6413003

[fsn33794-bib-0037] Redon, C. , Pilch, D. , Rogakou, E. , Sedelnikova, O. , Newrock, K. , & Bonner, W. (2002). Histone H2A variants H2AX and H2AZ. Current Opinion in Genetics & Development, 12(2), 162–169. 10.1016/s0959-437x(02)00282-4 11893489

[fsn33794-bib-0038] Saad, R. A. , & Mahmoud, Y. I. (2014). Ursodeoxycholic acid alleviates cholestasis‐induced histophysiological alterations in the male reproductive system of bile duct‐ligated rats. Reproductive Toxicology, 50, 87–97. 10.1016/j.reprotox.2014.10.011 25461907

[fsn33794-bib-0039] Said, R. S. , Mohamed, H. A. , & Kamal, M. M. (2019). Coenzyme Q10 mitigates ionizing radiation‐induced testicular damage in rats through inhibition of oxidative stress and mitochondria‐mediated apoptotic cell death. Toxicology and Applied Pharmacology, 383, 114780. 10.1016/j.taap.2019.114780 31618661

[fsn33794-bib-0040] Samarth, R. M. , & Samarth, M. (2009). Protection against radiation‐induced testicular damage in Swiss albino mice by Mentha piperita (Linn.). Basic & Clinical Pharmacology & Toxicology, 104(4), 329–334. 10.1111/j.1742-7843.2009.00384.x 19320637

[fsn33794-bib-0041] Silva, A. M. , Correia, S. , Casalta‐Lopes, J. E. , Mamede, A. C. , Cavaco, J. E. , Botelho, M. F. , Socorro, S. , & Maia, C. J. (2016). The protective effect of regucalcin against radiation‐induced damage in testicular cells. Life Sciences, 164, 31–41. 10.1016/j.lfs.2016.09.003 27620963

[fsn33794-bib-0042] Szumiel, I. (2015). Ionizing radiation‐induced oxidative stress, epigenetic changes and genomic instability: The pivotal role of mitochondria. International Journal of Radiation Biology, 91(1), 1–12. 10.3109/09553002.2014.934929 24937368

[fsn33794-bib-0043] Tian, Y. , Xiao, Y. , Wang, B. , Sun, C. , Tang, K. , & Sun, F. (2018). Vitamin E and lycopene reduce coal burning fluorosis‐induced spermatogenic cell apoptosis via oxidative stress‐mediated JNK and ERK signaling pathways. Bioscience Reports, 38(4), BSR20171003. 10.1042/BSR20171003 29273675 PMC6066653

[fsn33794-bib-0044] Toshimori, K. , Ito, C. , Maekawa, M. , Toyama, Y. , Suzuki‐Toyota, F. , & Saxena, D. K. (2004). Impairment of spermatogenesis leading to infertility. Anatomical Science International, 79(3), 101–111. 10.1111/j.1447-073x.2004.00076.x 15453611

[fsn33794-bib-0045] Türk, G. , Ceribaşi, A. O. , Sakin, F. , Sönmez, M. , & Ateşşahin, A. (2010). Antiperoxidative and anti‐apoptotic effects of lycopene and ellagic acid on cyclophosphamide‐induced testicular lipid peroxidation and apoptosis. Reproduction, Fertility, and Development, 22(4), 587–596. 10.1071/rd09078 20353718

[fsn33794-bib-0046] Turner, T. T. , & Lysiak, J. J. (2008). Oxidative stress: A common factor in testicular dysfunction. Journal of Andrology, 29(5), 488–498. 10.2164/jandrol.108.005132 18567643

[fsn33794-bib-0047] Tuttelmann, F. , Ruckert, C. , & Ropke, A. (2018). Disorders of spermatogenesis: Perspectives for novel genetic diagnostics after 20 years of unchanged routine. Medizinische Genetik, 30(1), 12–20. 10.1007/s11825-018-0181-7 29527098 PMC5838132

[fsn33794-bib-0048] Velez, D. , & Ohlander, S. (2021). Medical therapies causing iatrogenic male infertility. Fertility and Sterility, 116(3), 618–624. 10.1016/j.fertnstert.2021.07.1202 34462096

[fsn33794-bib-0049] Wdowiak, A. , Skrzypek, M. , Stec, M. , & Panasiuk, L. (2019). Effect of ionizing radiation on the male reproductive system. Annals of Agricultural and Environmental Medicine, 26(2), 210–216. 10.26444/aaem/106085 31232047

[fsn33794-bib-0050] Williams, E. A. , Parker, M. , Robinson, A. , Pitt, S. , & Pacey, A. A. (2020). A randomized placebo‐controlled trial to investigate the effect of lactolycopene on semen quality in healthy males. European Journal of Nutrition, 59(2), 825–833. 10.1007/s00394-019-02091-5 31591650 PMC7058571

[fsn33794-bib-0051] Xu, A. , Wang, J. , Wang, H. , Sun, Y. , & Hao, T. (2019). Protective effect of lycopene on testicular toxicity induced by benzo[a]pyrene intake in rats. Toxicology, 427, 152301. 10.1016/j.tox.2019.152301 31568845

[fsn33794-bib-0052] Xu, C. , Xu, J. , Ji, G. , Liu, Q. , Shao, W. , Chen, Y. , Gu, J. , Weng, Z. , Zhang, X. , Wang, Y. , & Gu, A. (2019). Deficiency of X‐ray repair cross‐complementing group 1 in primordial germ cells contributes to male infertility. The FASEB Journal, 33(6), 7427–7436. 10.1096/fj.201801962RR 30998386

